# Mass Spectrometry-Based Lipidomics Reveals Differential Changes in the Accumulated Lipid Classes in Chronic Kidney Disease

**DOI:** 10.3390/metabo11050275

**Published:** 2021-04-27

**Authors:** Lukasz Marczak, Jakub Idkowiak, Joanna Tracz, Maciej Stobiecki, Bartłomiej Perek, Katarzyna Kostka-Jeziorny, Andrzej Tykarski, Maria Wanic-Kossowska, Marcin Borowski, Marcin Osuch, Dorota Formanowicz, Magdalena Luczak

**Affiliations:** 1 Department of Natural Products Biochemistry, Institute of Bioorganic Chemistry Polish Academy of Sciences, 61-704 Poznan, Poland; jakubidkowiak1@gmail.com (J.I.); mackis@ibch.poznan.pl (M.S.); 2 Department of Analytical Chemistry, Faculty of Chemical Technology, University of Pardubice, 532 10 Pardubice, Czech Republic; 3 Department of Biomedical Proteomics, Institute of Bioorganic Chemistry Polish Academy of Sciences, 61-704 Poznan, Poland; joanna.a.tracz@gmail.com; 4 Department of Cardiac Surgery and Transplantology, Poznan University of Medical Sciences, 61-001 Poznan, Poland; bperek@ump.edu.pl; 5 Department of Hypertension, Angiology and Internal Disease, Poznan University of Medical Sciences, 61-001 Poznan, Poland; kostkajeziorny@gmail.com (K.K.-J.); tykarski@o2.pl (A.T.); 6 Department of Nephrology, Transplantology and Internal Medicine, Poznan University of Medical Sciences, 60-355 Poznan, Poland; wanic.kossowska.maria@gmail.com; 7 Institute of Computing Science, Poznan University of Technology, 60-965 Poznan, Poland; Marcin.Borowski@cs.put.poznan.pl; 8 Department of Molecular and Systems Biology, Institute of Bioorganic Chemistry Polish Academy of Sciences, 61-704 Poznan, Poland; mosuch@ibch.poznan.pl; 9 Chair and Department of Medical Chemistry and Laboratory Medicine, Poznan University of Medical Sciences, 60-806 Poznan, Poland; doforman@ump.edu.pl

**Keywords:** chronic kidney disease, cardiovascular disease, lipid profiling, lipidomics, mass spectrometry

## Abstract

Chronic kidney disease (CKD) is characterized by the progressive loss of functional nephrons. Although cardiovascular disease (CVD) complications and atherosclerosis are the leading causes of morbidity and mortality in CKD, the mechanism by which the progression of CVD accelerates remains unclear. To reveal the molecular mechanisms associated with atherosclerosis linked to CKD, we applied a shotgun lipidomics approach fortified with standard laboratory analytical methods and gas chromatography-mass spectrometry technique on selected lipid components and precursors to analyze the plasma lipidome in CKD and classical CVD patients. The MS-based lipidome profiling revealed the upregulation of triacylglycerols in CKD and downregulation of cholesterol/cholesteryl esters, sphingomyelins, phosphatidylcholines, phosphatidylethanolamines and ceramides as compared to CVD group and controls. We have further observed a decreased abundance of seven fatty acids in CKD with strong inter-correlation. In contrast, the level of glycerol was elevated in CKD in comparison to all analyzed groups. Our results revealed the putative existence of a functional causative link—the low cholesterol level correlated with lower estimated glomerular filtration rate and kidney dysfunction that supports the postulated “reverse epidemiology” theory and suggest that the lipidomic background of atherosclerosis-related to CKD is unique and might be associated with other cellular factors, i.e., inflammation.

## 1. Introduction

Chronic kidney disease (CKD) represents a kidney function disorder, which lasts at least three months and results from progressive damage of nephrons [1,2]. Despite the recognition of CKD as a major health problem worldwide, the awareness among patients and healthcare providers remains low [3]. CKD diagnosis is mainly based on decreased value of estimated glomerular filtration rate factor (eGFR), abnormal urinary albumin-to-creatinine ratio or occurrence of proteinuria [2,3].

Patients with CKD are at increased risk of cardiovascular diseases (CVD) [4]. The first stages of CKD (CKD1-2) reveal a mild reduction in kidney function and initial symptoms of CVD. Patients with stage 5 (CKD5) develop an end-stage renal disease (ESRD) and advanced CVD. Hypertension is found in about 70% of CKD and 80–90% of end-stage renal disease (ESRD) patients [5,6]. At early CKD stages, the likelihood of developing CVD increases two to three-fold due to deterioration of kidney function [6,7]. Furthermore, ESRD patients are at 20 to 1000-times higher risk of CVD as compared to the general population [8]. Moreover, the mortality rate related to cardiovascular events in CKD is almost three times higher than that for individuals without kidney dysfunction [4,9]. In classical CVD, dyslipidemia is recognized as one of the initial steps of atherosclerosis development resulting in subsequent cardiovascular complications. Elevated concentration of total cholesterol, followed by an increase in the atherogenic fraction of low-density lipoprotein (LDL) components, along with a low concentration of anti-atherogenic high-density lipoproteins (HDL) are crucial during atherosclerosis development [10]. However, in dialyzed CKD patients, an inverse association between cholesterol level and mortality, known as “reverse epidemiology”, has been reported [11,12,13]. The unique character of premature atherosclerosis in CKD has been unceasingly suggested [14,15,16,17]. Moreover, it has been postulated that the inverse relationship between cholesterol level and mortality in dialyzed CKD patients may be caused by several cholesterol-lowering factors, including malnutrition and systemic inflammation [18].

In our previous studies, we have shown that in CKD-related atherosclerosis (CKD-A) patients’ abnormalities in the accumulation of proteins involved in lipid transport and metabolism in plasma can be observed as compared to healthy people and persons with classical CVD without kidney dysfunction [19]. Consequently, a detailed study of lipidome alterations in CKD-A patients seemed worth pursuing. In CKD, the use of lipidomic profiling has been largely limited to the analysis of selected lipids, like cholesterol, its esters, triacylglycerols, and biochemical assemblies, e.g., HDL and LDL fractions [20]. However, in recent years, different accumulation of phospholipids and serum sulfatides in CKD patients, as well as first lipid biomarkers accompanying progression to ESRD, have been reported [20,21]. In these studies, experimental groups consisting mainly of patients with kidney dysfunction compared to age-matched controls have been considered. In turn, changes in lipid accumulation in patients with classical CVD, nonrelated to kidney dysfunction, have been investigated using different analytical approaches [22,23,24]. While lipid composition changes were observed in many inflammatory diseases, the alterations in CKD-A patients have been less assessed. To the best of our knowledge, lipid profiling in CKD-A and the association between CKD-A and dyslipidemia development remain undefined. Thus, detailed studies on lipidome in patients with CKD-A are of clinical importance.

The lipidomic approach relying on mass spectrometry has been frequently used in CVD investigations [22,23,24,25,26]. At present, direct infusion mass spectrometry, called “shotgun lipidomics”, and liquid chromatography-mass spectrometry (LC-MS) analysis constitute the most robust analytical approaches [27,28]. The interest concerning the high-throughput, precisely-reproducible and comprehensive shotgun approach in blood plasma lipidome screening is growing [27]. Moreover, new and improved protocols for shotgun lipidomics have been recently introduced, yielding unique lipid identification and data handling tools. These procedures cover method for plasma lipid extraction, analysis of obtained fractions with high-resolution mass spectrometers or triple quadrupoles equipped with a specific type of nano-electrospray ion source (nanoESI), but also bioinformatics and statistical typing of lipidomic signature [27,29,30].

This study focuses on plasma lipid profile alterations resulting from CVD development-related and nonrelated to CKD. We applied a shotgun lipidomics analytical approach to analyze plasma lipidome composition in two groups of CKD patients with different severity of the disease and underlying atherosclerosis, and a group of patients with the advanced stage of CVD but without any kidney dysfunction. Healthy volunteers without CKD and CVD were included in the study as a reference group. Furthermore, we utilized the analysis of lipid precursors and components using GC-MS. Such studies, based on complementary mass spectrometry approaches fortified with standard medical tests may shed light on the molecular mechanisms associated with atherosclerosis, related or nonrelated to CKD.

## 2. Results

This study investigated the disease-related differences in the lipidome of patients with atherosclerosis either related or nonrelated to CKD, and healthy volunteers. To achieve it, total lipid extracts from plasma were analyzed by the screening lipidomics approach. Further lipid blood measurement was conducted using standard medical examinations (Table 1). Finally, analysis of the selected precursors and components of lipids was performed. Since the composition of analyzed experimental groups slightly differed, we statistically evaluated if the identified lipids were related to statin, blood pressure, anticoagulant treatment, or gender. Such compounds were not identified in our study.

Laboratory tests have revealed that the concentration of total cholesterol, LDL and HDL fraction was significantly decreased in the CKD5 group, and exhibited the lowest levels as compared to other patients’ groups and healthy volunteers (HVs). The highest concentration of total cholesterol and LDL fraction has been measured in the CKD1-2 group. The levels of TAGs (triacylglycerols) were similar in both, CKD5 and CKD1-2, and revealed evident differences as compared to HVs and CVD group. CKD5 group displayed the lowest concentration of HDL in comparison to others. For this group, the highest concentration of C-reactive protein (CRP) was characteristic. Patients from CKD5 and CVD groups revealed a similar level of progression of atherosclerosis and CVD, however, all the above lipids measurements differed among these groups. The parameters presented in Table 1 served as variables for correlation analyses. The negative correlation between eGFR and CRP (r = −0.82) and glucose was demonstrated (r = −0.59). As expected, total cholesterol also revealed a positive correlation with LDL fraction (r = 0.58). However, HDL positively correlated with eGFR (r = 0.68) and negatively with glucose level (r = −0.53) and CRP (r = −0.51). The details of correlation analyses are presented in Table S1.

### 2.1. Lipid Classes Analysis Using Shotgun Approach

**Figure 1:**
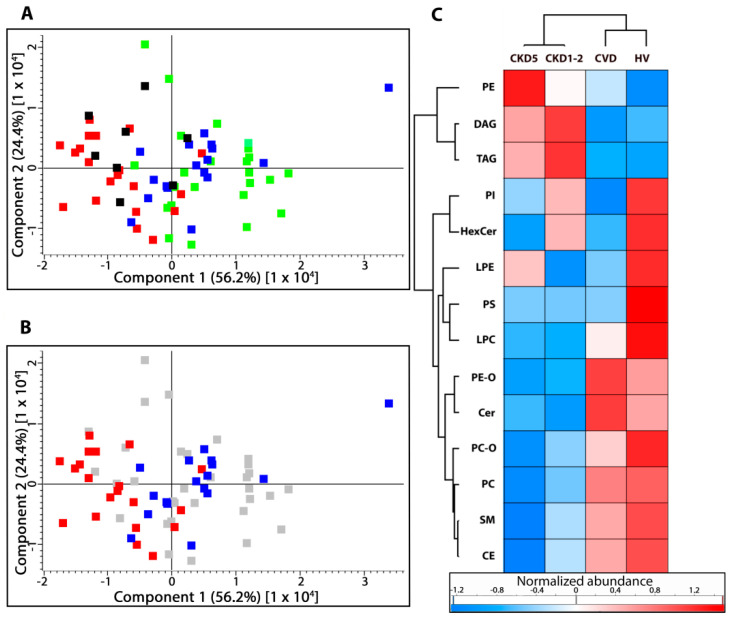
Unsupervised principal component analysis (PCA) performed on the total number of lipids identified in (**A**) stage 5 chronic kidney disease (CKD5) (red), cardiovascular disease (CVD) (blue), stage 1 and 2 chronic kidney disease (CKD1-2) (black), and healthy volunteers (HVs) (green) experimental groups. (**B**) To highlight the observed differences, only CKD5 (red) and CVD (blue) are marked. Other groups are presented in grey. (**C**) Hierarchical clustering analysis of identified lipid classes. PC, PC-O- phosphatidylcholines; PE, PE-O—phosphatidylethanolamines; PI—phosphatidylinositols; LPC—lysophosphatidylcholines; LPE—lysophosphatidylethanolamines; PS—phosphatidylserines; SM—sphingomyelins; Cer—ceramides; HexCer—hexosylceramides; TAG—triacylglycerols; DAG—diacylglycerols; CE—cholesterol and cholesteryl esters.

Utilizing plasma lipid profiling with a mass spectrometry approach, the presence of 14 lipid subclasses was revealed, including phosphatidylcholines (PC and PC-O), phosphatidylethanolamines (PE and PE-O), phosphatidylinositols (PI), lysophosphatidylcholines (LPC), lysophosphatidylethanolamines (LPE), phosphatidylserines (PS), sphingomyelins (SM), ceramides (Cer), hexosylceramides (HexCer), triacylglycerols (TAGs), diacylglycerols (DAG), and cholesterol/cholesteryl esters (CE). At first, the overall composition of lipid classes was compared. For this purpose, the average accumulation of each class of lipids was calculated and the data were scrutinized by one-way ANOVA/Kruskal–Wallis and t-test/U-Mann–Whitney tests. Nine out of 14 classes of lipids (CE, LPC, PE-O, Cer, PC, TAG, PS, LPE, and SM) were significantly altered amid the studied groups and revealed the false discovery rate (FDR) adjusted *p*-values below 0.05. HVs and CKD5 represented the most differentiating groups and exhibited the altered accumulation of all lipid classes. Details of the analysis are presented in Table S2. Principal component analysis encompassing all lipid species confirmed this observation and revealed that CKD5 and HVs varied most significantly from each other and the other groups (Figure 1A). Additionally, CKD5 and CVD groups demonstrated statistically significant differences in the accumulation of six classes of lipids (Figure 1AB). PCA did not separate HV and CKD1-2 groups (Figure 1A, marked in black (CKD1-2) and green (HVs). Observed differences were further confirmed by hierarchical cluster analysis (HCA, Figure 1C), in which the abundances of particular lipid classes in the studied groups can be observed. HCA merged two studied groups of CKD (CKD5 and CKD1-2) into one cluster, while the second cluster was represented by HV and CVD groups. HCA also revealed the upregulation of TAGs, DAGs, and PEs in both CKD groups and downregulation of CE, SM, PC, PE-O, and Cer as compared to CVD and HV cluster.

The partial least squares discriminant analysis (PLS-DA) also confirmed differences observed between HVs, CVD, CKD1-2, and CKD5, as these groups separate from each other in the presented score plot (Figure S1).

**Figure 2:**
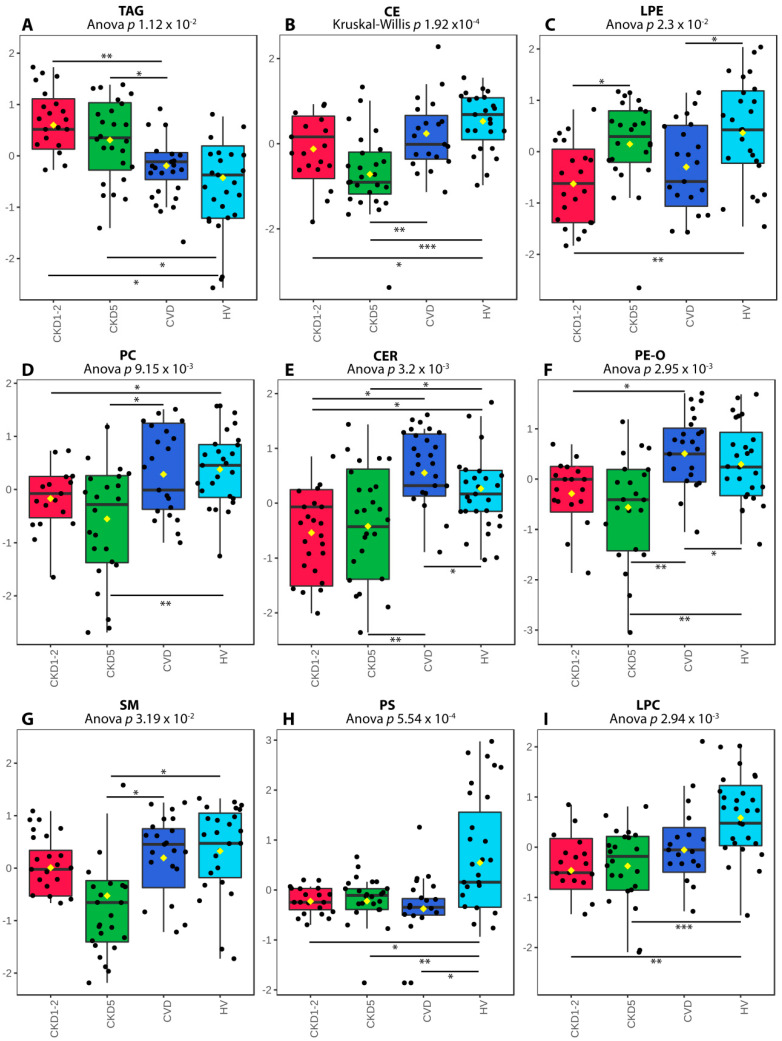
Differentially accumulated lipid classes sorted according to Anova/Kruskal–Wallis *p*-values: (**A**) triacyloglycerols (TAG). (**B**) cholesterol and cholesterol esters (CE). (**C**) lysophosphatidylcholines (LPE). (**D**) phosphatidylcholines (PC). (**E**) ceramides (CER). (**F**) phosphatidylethanolamines (PE-O). (**G**) sphingomyelins (SM). (**H**) phosphatidylserines (PS). (**I**) lysophosphatidylcholines (LPC). A box on a plot presents interquartile ranges and medians. The black dots correspond to the normalized accumulation of lipid classes in all samples. The notch indicates the 95% confidence interval around the median of each group. The mean level is indicated with a yellow diamond. Bars and asterisks show the results of U-Mann–Whitney/*t*-test: * *p* < 0.05, ** *p* < 0.01, *** *p* < 0.001; *n* = 25 in each group.

Details of this analysis are presented in Figure 2. Both CKD1-2 and CKD5 disclosed the highest level of TAGs as compared to HVs and CVD group (Figure 2A), which corroborated the results of standard medical examinations. In contrast, the lowest level of CEs was identified in the CKD5 group (Figure 2B), which confirmed the results of standard lipid profiling and suggested the correctness of performed MS/MS measurements. CKD1-2 and CKD5 groups differed in LPE accumulation only (Figure 2C). However, we also demonstrated a decrease in accumulation of PC, Cer, PE-O, and SM, in CKD5 patients in comparison to CVD (Figure 2D–G). Furthermore, CKD1-2 and CVD groups showed statistically significant differences in TAG, Cer and PE-O accumulation (Figure 2A,E,F). Downregulation of PSs and LPC was further shown in all patients’ groups compared to HVs (Figure 2H,I).

**Figure 3:**
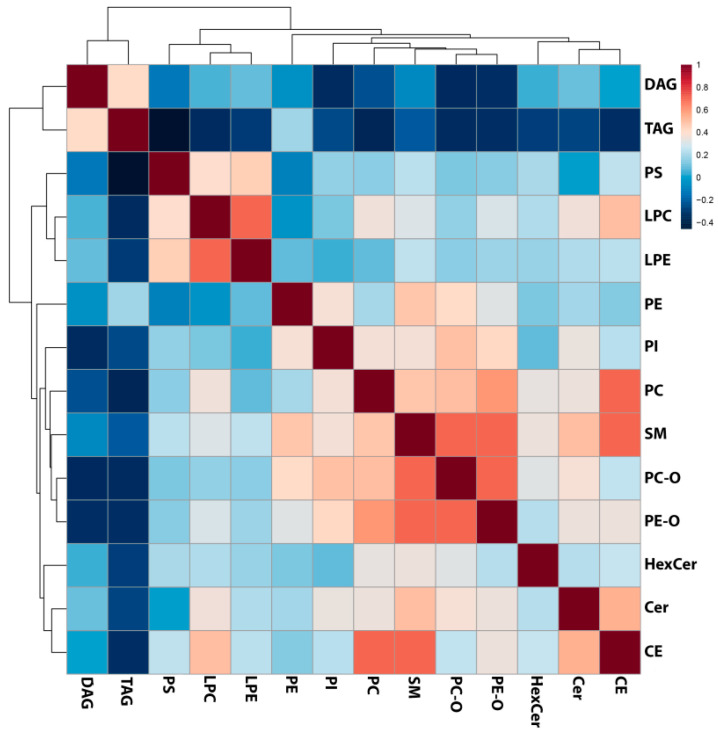
Results of the Pearson’s correlation analysis performed for all identified lipid classes and presented as a heat map with the corresponding hierarchical tree. Color saturation corresponds to the value of Pearson’s correlation coefficient.

To reveal the possible relationship between analyzed classes of lipids, in the next step, fourteen classes of lipids were correlated using Pearson’s correlation, and the resulting matrix was visualized as a heat map (Figure 3). Correlation analysis revealed that lipids belonging to similar classes were associated with each other, i.e., PC positively correlated with PC-O (r = 0.49) and PE-O (r = 0.59), and LPE with LPC (r = 0.69). Moreover, a Pearson’s correlation test revealed that SMs were correlated with PE-O (r = 0.66), PC-O (r = 0.68), CE (r = 0.61), and Cer (r = 0.49). Additionally, CE group correlated with PC (r = 0.65). The only inverse correlation was found between TAG and PS (r = −0.51). The details of correlation analyses are presented in Table S3.

The correlation analyses were also performed utilizing the analyzed classes of lipids and medical parameters presented in Table 1. Correlation between CE group and total cholesterol level measured in medical examinations corroborated the obtained results (r = 0.495). Pearson’s correlation test revealed that CE also correlated with CRP (r = 0.46), and eGFR (r = 0.47). Additionally, LPC group negatively correlated with glucose levels (r = −0.403). Other parameters demonstrated only medium or weak correlation (r = 0.23–0.40; Table S3).

### 2.2. Lipid Species Analysis Utilizing Shotgun Approach

In the next step, the changes in the particular lipid species abundance were found. High-throughput shotgun analysis of blood plasma allowed for the identification and quantification of 253 lipid species in total, including 24 Cers, 10 CEs, 12 DAGs, 45 TAGs, 14 LPCs, 6 LPEs, 24 PC-Os, 12 PIs, 14 PE-Os, 21 PEs, 4 PSs, and 28 SMs, 9 HexCers and 30 PCs. All of them were included in the subsequent statistical surveys and a particular lipid was considered to be differentially accumulated between at least two groups if the difference was statistically significant (*p* < 0.05), at the fold change of +/−1.5. The list of differentially accumulated lipids sorted according to appropriate statistical tests is presented in Table S4. The statistical comparison of quantitative lipidomic data revealed 61 lipid species differentiating all experimental groups (ANOVA/Kruskal–Wallis, FDR-adjusted *p*-value < 0.05). Ninety-one differentially accumulated lipid species were identified when separate comparisons were performed for all groups using *t*-test or U-Mann–Whitney test. These analyses demonstrated an altered accumulation of 28 and 50 lipid species when HVs were compared to CKD1-2 and CKD5 groups, respectively. Twenty-three lipid species differentiated HVs and CVD group. Differential accumulation of 24 and 17 lipid species, was revealed when CKD1-2 was compared to CVD and CKD5 group. Twenty-three differentially accumulated lipids were identified in CKD5 vs. CVD comparison.

**Figure 4:**
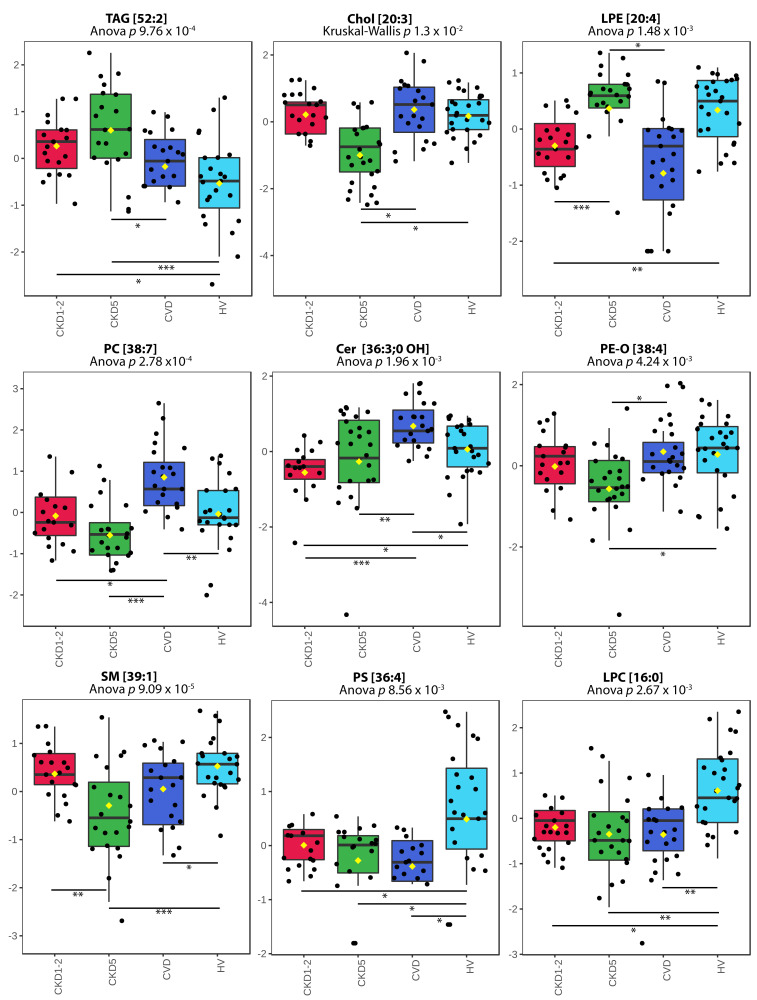
Abundances of representative species for all classes of lipids. (**A**) TAG [52:2]. (**B**) Chol [20:3]. (**C**) LPE [20:4]. (**D**) PC [38:7]. (**E**) CER [36:3:0, OH]. (**F**) PE-O [38:4]. (**G**) SM [39:1]. (**H**) PS [36:4]. (**I**) LPC [16:0]. A box on plot presents the interquartile ranges and medians. The black dots correspond to the normalized accumulation of selected lipid species in all samples. The notch indicates the 95% confidence interval around the median for each group. The mean level is indicated with a yellow diamond. Bars and asterisks show the results of U-Mann–Whitney/*t*-test: * *p* < 0.05, ** *p* < 0.01, *** *p* < 0.001; *n* = 25 in each group.

The high accumulation of TAGs characterized both CKD1-2 and CKD5 and considerably differentiated these groups from the other ones (Figure 4). Accumulation of 14 TAGs (all differential ones) was on average 2.03 and 2.13 times higher in CKD1-2 or CKD5 than in CVD group. Furthermore, all identified TAGs were also upregulated when CKD1-2 or CKD5 groups were compared to HV, with average fold changes of 1.78 and 1.67, respectively. The exemplary TAG [52:2] profile is presented in Figure 4A. We also identified two DAGs: [34:3] and [32:0], with similar abundance profiles as TAGs. The results obtained for specific CEs were consistent with the data gained for all classes of lipids (Figure 4B). Five differentially accumulated CE species were identified and found to be downregulated in the CKD5 group. The respective fold changes ranged between 0.3 and 0.62 in CKD5 vs. HV comparison and 0.52–0.62 in CKD5 vs. CVD comparison. The representative CE [20:3] profile is presented in Figure 4B. The robust difference in the accumulation of particular lipid species was identified when HVs and CKD5 groups were compared. The levels of 49 different molecules were found to be altered, including five downregulated CEs in CKD5 as compared to HV. In addition, seven differentially regulated SM (Figure 4G), seven LPC (Figure 4I), five PC (Figure 4D), three PC-O, three PE-O (Figure 4F), two PS (Figure 4H), two Cer, one PI and one LPE were decreased in their abundance in CKD5, in comparison to HVs. On the other hand, seven upregulated TAGs (Figure 4A), two DAGs and five PE were identified in CKD5. A similar difference, but to a lower extent, was identified when HV was compared with CKD1-2. In particular, nine TAGs (Figure 4A) and one DAG were found to be upregulated in CKD1-2, while one SM (Figure 4G), six LPCs (Figure 4I), two PS (Figure 4H), two PCs (Figure 4D), one PE-O, two Cer, two LPE (Figure 4C), one CE and one PI were downregulated in CKD1-2 in comparison to HV group.

Subsequently, we compared two groups of CKD patients. We have observed that CKD1-2 and CKD5 patients displayed a similar lipid profile but differed in the accumulation of PC/PC-O, which were upregulated, and PEs that were found to be downregulated in CKD1-2 vs. CKD5 comparison.

Furthermore, the differences between CKD and CVD patients were disclosed. Apart from the upregulation of TAGs and downregulation of CEs in CKD compared to CVD, also two DAGs revealed the same directionality of change. All identified differential phospholipid species, i.e., nine PCs, three PE-Os, one PC-O, two PEs were downregulated in both CKD groups (as compared to CVD). Additionally, three SM and three Cer accumulated at significantly lower levels in both CKD groups.

**Figure 5:**
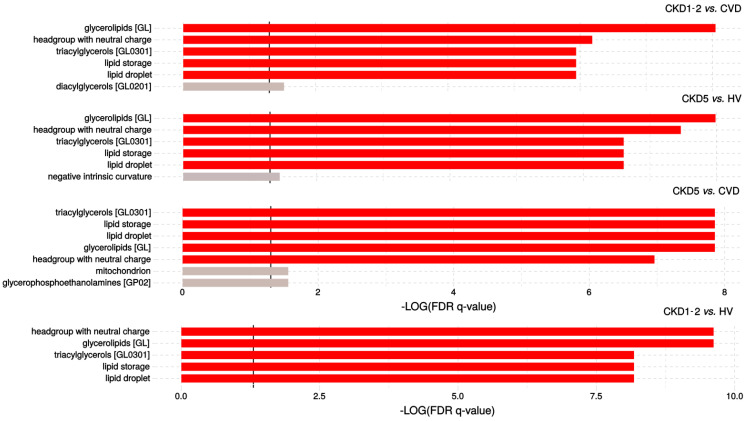
LION-term enrichment analysis of CKD1-2 vs. CVD, CKD5 vs. HV, CKD5 vs. CVD and CKD1-2 vs. HVs presented in the “ranking mode”. The gray vertical lines indicate the threshold of –log10 False discovery rate (FDR)-corrected *p*-value 0.05 (*q*-value 1.3). Red bars present terms with FDR-corrected *p*-value < 0.01 (*q* > 2).

In the next step, the enrichment analysis was performed in LION software to show a possible overrepresentation of functional terms related to lipid functions, cellular components, or lipid classification (Figure 5). Lipid identifiers were ranked by logarithmic transformation and fold-change values.

Seven different features including lipid storage and formation of lipid droplets, were overrepresented according to FDR-corrected Kolmogorov–Smirnov *p*-value and statistically enriched in the CKD5 vs. CVD2 comparison. The results also suggested that triacylglycerols and glycerophosphoethanolamines are important in the differentiation of CKD5 and CVD patients. In the case of CKD1-2 vs. CVD comparison, similar results concerning functions in lipid storage have been found, but glycerolipids and lipids with neutral head groups were the main enriched group. Almost the same results were observed in comparison of CKD5 to HVs, and CKD1-2 vs. HV. Interestingly, no important enrichment was observed in the comparison of CVD to HV. The detailed information of this analysis is presented in Table S5.

**Figure 6:**
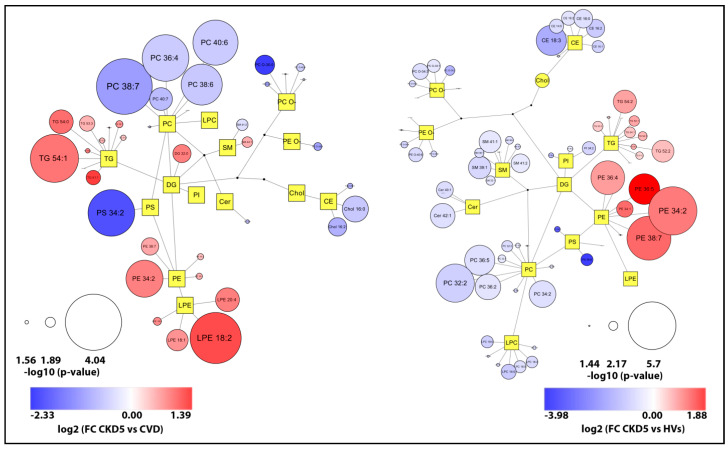
Network visualization of the most dysregulated lipid species found in CKD5 vs. CVD (left panel) and CKD5 vs. HV comparisons. Graphs express lipidomic pathways connections with clustering into individual lipid classes prepared using the Cytoscape software. Circles correspond to detected lipid species, the circle size articulates the significance according to Mann Whitney U test *p*-value, while the color gradient explains the degree of variations (red/blue) according to the calculated fold change.

To gain more functional insight, we further analyzed associations between identified classes of lipids (Figure 6). The plots illustrate overall changes observed in the lipidome of two the most significant group comparisons and revealed downregulation of PCs in CKD5 in comparison to CVD group and upregulation of PEs in CKD5 in comparison to HVs.

### 2.3. Analysis of Selected Lipid Precursor and Components Using GC-MS Profiling

**Figure 7:**
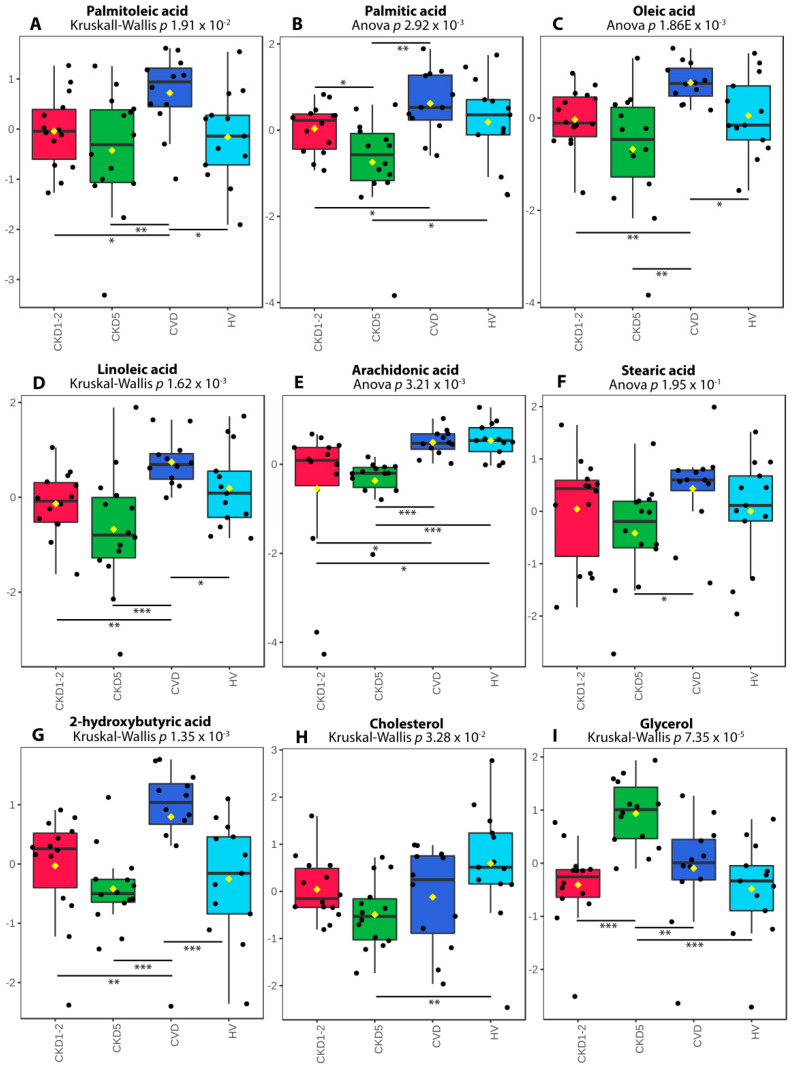
The abundance of differentially accumulated fatty acids, cholesterol and glycerol revealed in GC-MS analysis. (**A**) Palmitoleic acid. (**B**) Palmitic acid. (**C**) Oleic acid. (**D**) Linoleic acid. (**E**) Arachidonic acid. (**F**) Stearic acid. (**G**) 2-hydroxybutyric acid. (**H**) Cholesterol. (**I**) Glycerol. A box on plot presents interquartile ranges and medians. The black dots represent the normalized accumulation of molecules in all samples. The notch indicates the 95% confidence interval around the median of each group. The mean level is indicated with a yellow diamond. Bars and asterisks show results of U-Mann–Whitney/*t*-test: * *p* < 0.05, ** *p* < 0.01, *** *p* < 0.001; *n* = 14 in each group.

To gain more information about the observed changes, we subsequently chose the 14 most common precursors or components of lipids, including saturated, unsaturated fatty acids, glycerol, glyceric acid, and cholesterol, and performed the GC-MS metabolomic analysis utilizing separate cohorts. The low-molecular-weight compounds were quantified based on their peaks integrated from extracted-ion chromatograms (EIC). The results of this analysis are presented in Table S6. We have identified seven fatty acids, glycerol and cholesterol as differentiating lipid species among the analyzed experimental groups (Figure 7).

Data obtained for cholesterol corroborated the results of lipid profiling and standard medical tests, and revealed its lowest level in the CKD5 group (Figure 7H). However, a statistically significant change was only observed in CKD5 vs. HV comparison. All analyzed fatty acids revealed the lowest abundance in CKD5 plasma. However, substantial differences between particular groups of patients have also been detected (Figure 7A–G). For instance, CKD1-2 and CKD5 patients differed only in the level of palmitic acid (Figure 7B), although all fatty acids were significantly altered when CKD5 patients were compared to CVD ones (Figure 7A–G).

**Figure 8:**
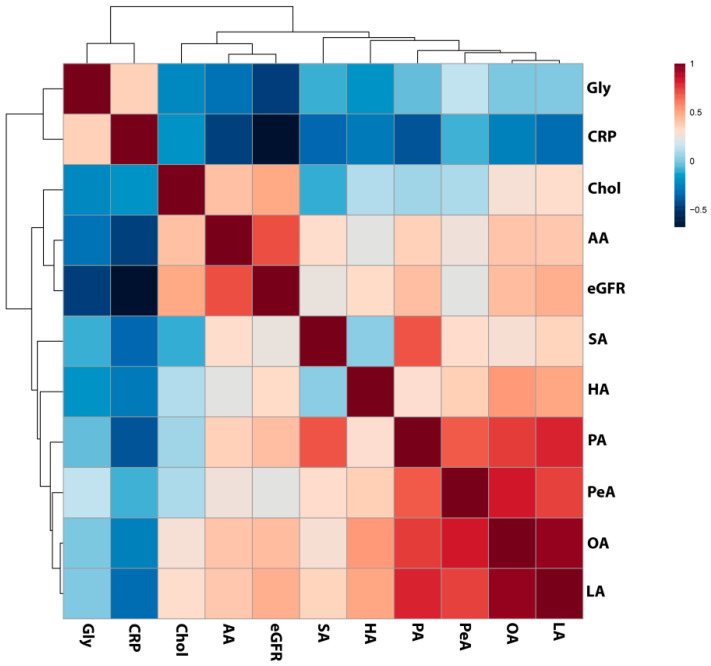
Results of the Spearman’s rank correlation test performed for compounds identified in GC/MS analyses as differentially accumulated between analyzed experimental groups and estimated glomerular filtration rate (eGFR) and C-reactive protein (CRP) level presented as a heat map with the corresponding hierarchical tree. Color saturation corresponds to the value of Spearman’s correlation coefficient. Gly—glycerol, Chol—cholesterol, AA—arachidonic acid, SA- stearic acid, HA—hydroxybutyric acid, PA—palmitic acid, PeA—palmitoleic acid, OA—oleic acid, LA—linoleic acid.

The opposite result was also disclosed—the CKD5 group revealed the highest abundance of this molecule, which differed significantly as compared to CVD, CKD1-2 and HVs (Figure 7I). Moreover, four identified fatty acids: linoleic, palmitic, palmitoleic and oleic acids strongly, positively correlated with each other, with ρ > 0.75 (Figure 8; Table S7). Palmitic acid strongly correlated with stearic acid, with ρ = 0.73. We also correlated the abundance of identified compounds with eGFR. The analysis revealed that lower eGFR corresponded to a lower abundance of arachidonic acid (ρ = 0.7), palmitic acid (ρ = 0.42), oleic acid (ρ = 0.43), linoleic acid (ρ = 0.47), and cholesterol (ρ = 0.49). On the other hand, glycerol showed an inverse relationship with eGFR (ρ = −0.46). Additionally, CRP revealed a correlation with arachidonic (ρ = −0.45) and palmitic acid (ρ = −0.40).

## 3. Discussion

The results of our previous proteome analyses showed that the accumulation of proteins involved in lipid metabolism and transport differed considerably in patients with CKD-A and atherosclerosis nonrelated to CKD, i.e., found in CVD patients [19]. In this study, we aimed to investigate changes in the blood plasma lipidome, in CKD and CVD patients. In routine clinical practice, lipid measurement based on the estimation of lipid concentration does not allow for the complex characterization of alterations in the plasma lipid profile. Human blood plasma lipidome can be characterized as highly diverse and thereby, more precise and accurate analysis needs to be performed to reveal the subtle abnormalities. The results of lipidome screening in CKD by mass spectrometry or hyphenated techniques have been published recently. For instance, Reis et al. analyzed LDL fractions in CKD patients and controls, and identified 352 lipid species. They demonstrated that the disease progression was associated with evident changes in lipid profile, while the results of clinical, biochemical tests might appear normal [20]. Using LC-MS several phosphatidylethanolamines and monoacylglycerols related to CKD progression were identified [21]. However, to the best of our knowledge, our study demonstrated for the first time the comparison between patients with atherosclerosis-related and unrelated to CKD.

Among CKD patients, unique dyslipidemia which affects CVD progression is frequently observed and characterized as resistant to standard interventions [31]. Elevated levels of triacylglycerols (TAGs) and normal or reduced HDL and LDL are often seen [2,12,31]. Mikolasevic et al. indicated that hypertriglyceridemia occurs in 40–60% of CKD1-4 individuals and 45% of hemodialyzed patients [32]. Previous studies demonstrated a link between decreased catabolism of TAGs-rich lipoproteins (i.e., VLDL, LDL, and chylomicrons), caused by downregulation of certain genes involved in lipid metabolism and transport, and higher accumulation of TAGs in the blood of patients with CKD [33]. Authors suggested that the presence of uremic toxins inhibits lipid processing enzymes and alters lipoprotein particles’ composition, which was considered to represent the main mechanisms of hypertriglyceridemia in patients with kidney dysfunction. Interestingly, the relation between impaired catabolism of TAG-carrying particles, the elevated abundance of TAGs, proteins participating in mentioned processes, and inflammation progression was suggested.

Therefore, the higher abundance of TAGs observed in CKD is not surprising. TAGs are esters derived directly from glycerol and fatty acid molecules. Interestingly, we observed a decreased abundance of seven fatty acids (commonly present in TAG structure) in CKD, especially in CKD5 patients, and four of them; palmitic, palmitoleic, oleic and linoleic, were strongly correlated with each other. In contrast, the level of glycerol was elevated in CKD5 in comparison to all analyzed experimental groups. Treatment with glycerol causes severe renal dysfunction, which includes renal structural abnormalities, renal oxidative stress, inflammation and apoptosis, and this molecule is frequently used to induce kidney injury in animal models [34,35,36]. However, a high level of endogenous glycerol in humans has not been observed so far. It is also noteworthy that arachidonic, palmitic, oleic and linoleic acid positively associated with renal function, whereas glycerol was inversely associated with eGFR and CRP. Fatty acids in kidney disease are sporadically described in the literature. Nevertheless, the correlation between the level of plasma fatty acids in CKD and disease progression and mortality has been previously demonstrated [37]. Stearic acid abundance was decreased in CKD patients, which is in line with our findings [38]. In this study, also oleic and linoleic acid were correlated with kidney dysfunction parameters, and the authors suggested an association with CKD. Furthermore, downregulation of oleic, linoleic and arachidonic acids was observed in hemodialyzed patients as compared to the control group [39]. Moreover, the relative abundance of saturated fatty acids and mono-unsaturated fatty acids was associated with lipid disorders and cardiomyopathy in CKD [39]. The positive relationship between saturated fatty acids accumulation and vascular calcification, the most common complications related to atherosclerosis progression in CKD, has also been reported [40]. Furthermore, the reduced level of 2-hydroxybutyric acid was observed in advanced renal patients [41]. The authors suggested that the presence of decreased level of 2-hydroxybutyric acid is related to the declining of H_2_S generation process, which was confirmed by the reduced expression of enzymes involved in these reactions. The downregulation of H_2_S-synthesis and related enzymes was also presented in an animal model of CKD [42] and in the circulating leukocytes of ESRD patients [43,44]. As an anti-inflammatory, anti-oxidant and anti-apoptotic compound, H_2_S has been associated with cardiovascular and kidney disease. Therefore, the differences in the CKD and CVD level of 2-hydroxybutyric acid, a metabolite related to H_2_S production, but also other identified fatty acids once again underlines the differences between molecular mechanisms underlying disease progression in CKD and CVD.

We have earlier suggested that dysregulated metabolism of pro-atherogenic and anti-atherogenic protein fractions of lipoproteins transporting primarily TAGs is far more advanced in CKD than patients with the classical cardiovascular disorder without kidney dysfunction [45]. Apolipoprotein B and apolipoprotein A1 are critical for lipoprotein formation, stability and clearance, while apolipoprotein A4 is involved in reverse cholesterol transport and plays an anti-atherogenic and antioxidative role. Our previous studies revealed the altered levels of these proteins in CKD as compared to HVs and CVD patients. Therefore, the impaired accumulation of TAGs can be a consequence of processes associated with lipoprotein particles’ changed composition.

Furthermore, low HDL levels were found in 35–50% of cases of CKD1-5 [32]. Our results concerning the clinical indices showed significantly decreased total cholesterol levels, LDL and HDL in CKD5 compared to others, despite advanced atherosclerosis and high incidence of cardiovascular events observed in this group. The lowest concentration of total cholesterol was observed for the CKD5 group, as revealed by three independent, complementary approaches: shotgun lipid profiling, standard medical tests and GC-MS quantification. Moreover, we also showed a correlation between the cholesterol level, and eGFR and thus demonstrated that the lower cholesterol level might be related to kidney dysfunction progression. The negative association between cholesterol levels and mortalities in dialyzed patients known as “reverse epidemiology” was postulated in a few studies [12,31,46,47]. Some studies suggested that LDL lowering therapy can relatively reduce the risk of mortality among renal failure patients [2]. In contrast, Kilpatrick et al. demonstrated that adjustments for inflammation and malnutrition led to equivocal results, and did not confirm the thesis of the increased mortality in the group of CKD5 patients with low LDL [47]. In our study, the inflammation process was significantly elevated in CKD5 patients as depicted by the level of C-reactive protein. Moreover, we have earlier demonstrated that proteins involved in inflammation exhibited more significant alterations in individuals with CKD-related atherosclerosis [48]. Therefore, severe malnutrition and inflammation development should be considered as the most critical factors responsible for CVD progression in CKD.

Previously, we have also demonstrated an absence of a correlation between the protein components of LDL and LDL level, and indicated that LDL might be more atherogenic in CKD-A vs. classical CVD patients [19]. Large differences in accumulation of many lipid species between CVD and CKD5 groups, with similar atherosclerosis progress, observed in the current study, strengthen our hypothesis that different molecular mechanisms are involved in atherosclerosis-related and nonrelated to CKD. Apart from TAGs, the highest differences in CKD5 vs. CVD comparison were observed for CEs, PCs, PE-Os, SMs and Cers. However, in contrast to TAGs, all of these classes revealed decreased abundance in CKD5 compared to CVD. Moreover, changes in abundances of many of these classes were strongly correlated with each other. It should be stated that the dialysis procedure itself shall not cause the loss of hydrophobic molecules, but the loss of some proteins like albumin, which may bind lipid particles, is possible [49]. Moreover, samples were collected before the hemodialysis session which excludes the influence of treatment.

It is worth mentioning that CKD5 and CVD groups of patients with similar symptoms and progress of atherosclerosis (based on medical records) differed in the accumulation of 23 from a total of 91 differentiating lipid species. On the other hand, the comparison between CVD and CKD1-2 groups, which are considerably different in the severity of atherosclerosis, also revealed 24 differentially accumulated compounds. However, distinct lipid species were identified as differentially accumulated in both comparisons. These observations support our hypothesis that the CVD acceleration mechanism may differ at initial and advanced CKD stages, in which the inflammation processes are more pronounced. Accumulation of 17 different lipid species differentiated CKD1-2 and CKD5 groups, and these compounds could serve as disease progression indicators in CKD.

A few studies have shown that sphingomyelin is associated with cardiovascular disease progression [50]. Moreover, CKD sphingomyelin was found to be significantly and directly associated with GFR decline [51]. Our results seem to be contrasting with them, however, studies comparing both groups, CKD and CVD at the same have not been conducted in this aspect.

The molecular complexity of the CKD-A should also be taken into account. Although precise relation(s) between differentially accumulated lipid species and genes or enzymes involved in lipid homeostasis was not investigated, we can refer to the other results. Some researchers suggested that significantly increased cellular accumulation of CEs and DAGs can be linked with the activation of the mitogen-activated protein kinase and protein kinase C pathways involved in the process of inflammatory milieu development [21]. Moreover, the intensified biosynthesis of particular PEs could confirm the protein kinase C activation, leading directly to the degradation of membrane components and influencing the downstream membrane-protein physiological processes [21]. Further research shall be conducted on the role of inflammation caused by progressive kidney damage and its influence on the process of cardiovascular disorder development.

Considering a functional analysis of results we should emphasize that lipidomics is still a novel strategy in system biology sciences and all the available tools are still in their early development. LION is as far as we know the only tool allowing for reasonably effective enrichment analysis of lipid species. Our results strongly suggest that the main function of lipids involved in the progression of CKD is lipid storage and formation of lipid droplets, it also shows that these lipids are mainly TAGs and DAGs, but also phospholipids of a different kind. As lipid droplets are reservoirs of cholesterols in mammalian cells, the dysregulation of their formation may be of important function especially in the development of atherosclerosis and it may explain why we observe these changes both in CKD and CVD patients.

The limitations of the current study are related to the identification of only a portion of the plasma lipidome. Another limitation is the lack of common and verified methods for validation of lipid analytes that we can utilize in, for instance, proteomic studies. Therefore, we supplemented the lipid profiling with standard medical assessments of lipoprotein fractions and GC/MS analysis of selected lipid components and precursors. Utilizing such complementary methods, we successfully validated the cholesterol levels in all analyzed experimental groups. The dietary habits of studied participants were not analyzed in this study. Nonetheless, the assumption about the dietary variation equal in all groups of individuals can be considered. Finally, an additional research in larger CKD and CVD cohorts is required to validate the findings presented in this small-scale study.

To the best of our knowledge, the study comparing lipid profiles of patients with atherosclerosis-related and nonrelated to CKD has not been presented before. These findings, combined with lipid precursors and components analysis and clinical indices, confirmed our previous hypothesis that atherosclerosis underlying CVD in CKD may be uniquely assessed, differentiating it from patients with classical CVD without kidney disease. The mechanism of atherosclerosis progression in CKD is not yet fully elucidated, and is likely of multifactorial origin. Based on our results, it seems plausible that atherosclerosis in patients with CKD is more considerable than in CVD, and is associated with inflammation. Malnutrition and increasing concentration of uremic toxins that influence lipid metabolism and transport and inhibit the enzymes involved in lipid homeostasis can act as acceleratory factors. Hence, the targeted screening of lipid classes engaged in the inflammation process and analysis of oxidized forms of metabolites is worth pursuing in future studies, to reveal the mechanisms beyond the progression of CVD in CKD. In summary, our findings support the importance of exploring the lipid profiles to elucidate the underlying molecular mechanisms and pinpoint the new lipid-lowering strategies in CKD-A.

## 4. Materials and Methods

### 4.1. Subject and Samples

The study has been approved by the Bioethics Committee at the Poznan University of Medical Sciences (resolution no. 926/16). Our study protocol conformed to the Ethical Guidelines of the World Medical Association Declaration of Helsinki. All participating individuals provided signed informed consent for inclusion. One hundred individuals divided into four groups of equal size were involved in the survey (Table 1). Individuals with diabetes mellitus, acute inflammatory processes, and malignant tumors were excluded from the study. To further characterize the studied groups, eGFR (calculated by the formula developed by Levey et al. [52]), body mass index (BMI), high sensitivity C-reactive protein (hsCRP), and the full lipid profile were determined.

The systolic and diastolic blood pressure was measured, and medical history was established in all studied groups. Healthy volunteers and patients were qualified to appropriate experimental groups based on obtained clinical measurements and medical history. According to the National Institute for Health and Care Excellence guidelines, CKD patients were divided into two groups, i.e., CKD1-2 and CKD5 [53]. The CKD1-2 group was restricted to 25 patients at the initial CKD stages (mean eGFR 70 mL/min/1.73 m^2^) with hypertension, hyperlipidemia, diagnosed with nonobstructive coronary artery disease (CAD)—confirmed by coronarography, but without cardiovascular events in their medical history. The CKD5 and CVD groups included 25 patients each, displaying advanced atherosclerosis confirmed by coronarography and clinically manifested as severe CAD, with a history of at least one acute cardiac incident and/or status after the vascular intervention. The factor differentiating these two groups was the status of kidney function, the CKD5 patients with mean eGFR 6 mL/min/1.73 m^2^ represented ESRD patients treated with hemodialysis for at least two years, three times per week. Subjects from the CVD group exhibited normal kidney function with mean eGFR 96 mL/min/1.73 m^2^. The last group, which served as a control group, consisted of 25 age-matched healthy volunteers (HVs) with a mean eGFR of 102 mL/min/1.73 m^2^.

A closed monovette system containing EDTA was applied for peripheral blood collection. Plasma samples were stored at −80 °C until analysis.

### 4.2. Lipid Extraction Method

Lipid separation was carried out according to the MTBE extraction protocol proposed by Matyash and co-workers [29]. Briefly, 20 μL of each plasma sample was diluted with 80 μL of water in glass tubes with teflon-coated caps. Subsequently, 10 μL of methanolic solution of labeled standard (16:0-d31-18:1 PC, 1µg, from Avanti lipids), 750 μL of methanol and 2.5 mL of MTBE were added, and the mixtures were vortexed for 1 h at RT. Phase separation was induced by the addition of 625 μL of water. Upon 10 min incubation at RT, the mixtures were centrifuged at 1000× *g* for 10 min, 2 mL of upper organic phases were then collected and dried under a nitrogen stream at 37 °C. Lipids were resuspended prior to analysis in 500 μL of MS-mix buffer containing 7.5 mM ammonium acetate, in chloroform, 2-propanol and methanol (1:2:4 *v*/*v*/*v*).

### 4.3. Mass Spectrometry Analysis of Lipid Fractions (Shotgun-Based Lipidomics)

Lipid profiling of blood plasma samples was performed using Q-Exactive Orbitrap mass spectrometer (Thermo Fisher Scientific, Bremen, Germany) equipped with TriVersa NanoMate robotic nanoflow ESI ion source (Advion BioSciences ltd., Ithaca, NY, USA). The nanoelectrospray chips with nozzles’ diameter of 5.5 μm were used to obtain stable ion spray, and Chipsoft software (ver. 8.3.1.1018, Advion BioSciences ltd., Ithaca, NY, USA) was applied to control ESI ion source. The system was calibrated using Pierce™ LTQ Velos ESI Positive Ion Calibration Solution (Thermo Fisher Scientific, Rockford, IL, USA). 10 μL of the sample was infused directly into the mass spectrometer, and after ion current stabilization, data were acquired for 10 min. The source was operated at a gas pressure of 1.25 psi, and the optimum ionization voltage was set to 1.05 kV. Briefly, MS data were acquired in positive ion mode within the range of *m*/*z* 300–1500 at the resolution of 140,000 (at *m*/*z* 200, FWHM). Automatic gain control was set to a target value of 3.0 × 10^6^, and ion injection time (IT) at 100 ms. Targeted MS/MS experiments were carried out using higher-energy collisional dissociation mode (HCD) to assist in the identification of lipid species.

In order to control the quality of measurements, we have used pooled plasma samples derived from all samples as well as pools prepared for each group of patients separately. These samples were injected following every 8 samples in sequence. The reproducibility of the biological and technical replicates was assessed by the scatter plotting and the correlation coefficient determined based on the signal intensities. Samples with Pearson’s correlation coefficients above 0.9 were considered as those with good quality suitable for further analysis.

### 4.4. Data Processing and Identification of Lipid Species

Raw MS data were converted into mzXML format and further processed with LipidXplorer software (ver. 1.2.7, Max Planck Institute of Cell Biology and Genetics, Dresden, Germany) [54]. Profiles were obtained by averaging 8 min of recorded mass spectra, from the second minute to the ninth minute within ten minutes of sample delivery time. The first 60 s of sample injection was allowed for electrospray and analyte flow stabilization, based on total ion current (TIC) variation. Subsequently, an alignment procedure was performed to match the related peaks within the whole dataset. Defined signals in mass spectra for further processing and consideration were selected by setting the threshold value to 1500 (S/N ~3). The identification of lipid species was performed following Graessler et al. [25]. The LipidXplorer software was used, and MFQL files for the identification of lipids in human blood plasma were supplied from the MFQL library (available at: https://wiki.mpi-cbg.de/lipidx/MFQL_library, access on 12 March 2020). Particular lipid species were identified based on accurately determined masses, i.e., mass accuracy higher than 5 ppm in MS+ (the top-down lipidomics approach). The fragmentation patterns of selected lipids were additionally analyzed for the presence of specific signals derived from headgroups or bases, in order to confirm the subclass annotations: for LPC, PC and SM at *m*/*z* 184.07—phosphocholine headgroup, for CE at *m*/*z* 369.35—cholesterol base, and for ceramides at *m*/*z* 264.26—for the sphingosine d18:1 backbone; for PE and PS the neutral losses were observed of 141.02 Da (phosphoethanolamine), and of 185.01 Da for phosphoserine, respectively (https://lipidomics-standards-initiative.org/resources/lipid-class-specific-fragments, access on 9 April 2021). The annotations were additionally compared with the data from the LipidMaps and the Human Metabolome Database library. Following the recommendations by MSI (Metabolite Standard Initiative), the compound names were assigned within each class according to the second and third level of identification. The second level indicates the putatively annotated compounds based on physicochemical properties and/or spectral similarity with public/commercial spectral libraries. The third level designates the putatively characterized compound classes based on characteristic physicochemical properties of a chemical class of compounds, or by spectral similarity to known compounds of a chemical class. Both levels of identification designate them as putatively annotated compounds, according to Sumner and co-workers [55].

All identified lipids were analyzed separately, and average abundance for each lipid class was also calculated. The average accumulation was based on the normalized accumulation of all identified lipids that belonged to the same class.

### 4.5. Functional Analysis

LION (Lipid Ontology—http://www.lipidontology.com/, access on 9 April 2021) tool was used for enrichment analysis and lipids’ association with pathways [56]. For this purpose, tables with quantitative information were prepared according to instruction present on the website and “Ranking Mode” for data pretreatment was used. Lipid identifiers were ranked by logarithmic transformation and fold-change values. The 1-tailed Kolmogorov–Smirnov tests were used as “global” statistics to assess enrichment of LION-terms over a ranked (by “local” statistics) list of lipids.

In addition to the results from LION, plots in which the core structure reflects basic (simplified) lipid metabolism were prepared. The up- and downregulated lipids in a target group were presented as circles, where the size is related to the statistical significance (Mann Whitney U test *p*-value), and the color to the fold change, respectively. The plots capture overall changes observed in the lipidome of one group, compared to control (or another group). Graphs were prepared using the Cytoscape software (http://www.cytoscape.org, access on 9 April 2021).

### 4.6. GC-MS Analysis of Plasma Samples for Identification of Selected Lipid-Related Compounds

Twenty-five microliter aliquots of plasma samples were deproteinized with 4 volumes of cold methanol and stored in a freezer for 20 min at −20 °C. Samples were centrifuged at 11,000× *g* for 5 min, and supernatants were transferred to Eppendorf tubes. Samples were then dried in a vacuum centrifuge, resuspended in 20 µL of methoxyamine hydrochloride (20 mg/mL in dry pyridine), and vortexed in a thermomixer for 1.5 h at 37 °C. Fifty µL of MSTFA *(N*-Methyl-*N*-(trimethylsilyl)-trifluoroacetamide) was then added to each sample, vortexed for 30 min at 37 °C and centrifuged at 11,000× *g* for 10 min. From each sample, 50 µL of the mixture was subsequently transferred to a conical glass vial prior to GC separation. One-μL aliquots of the derivatized samples were injected using PTV-injection in the splitless mode. Metabolites were analyzed using GC-MS system (TRACE 1310 GC oven with TSQ8000 triple quad MS; Thermo Fisher Scientific, Rockford, IL, USA), equipped with DB-5MS column (30 m × 0.25 mm × 0.25 µm; J&W Scientific, Agilent Technologies, Santa. Clara, CA, USA). Chromatographic separation conditions in gradient mode were kept as follows: 70 °C for 2 min, followed by 10 °C/min up to 300 °C, at 300 °C (10′). PTV injector was used for sample injection with a temperature gradient from 40 to 250 °C, column interface was kept at 250 °C, and source temperature at 250 °C. The ion source operated in 50–850 *m*/*z* range, in EI positive mode, and electron energy was set to 70 eV. As TMS (trimethylsilyl) derivatives, the compounds were identified after comparisons of registered mass spectra with those present in the NIST library. Identified compounds were quantified, and extracted ion chromatograms (EIC) of selected *m/z* values, corresponding to specific compounds, together with specific retention times for given compounds, were used. First, the unique masses were chosen based on library spectra, and EIC peaks were integrated to measure their areas, and respective values were exported into tables. Furthermore, the total ion current was measured by integration of all TIC peaks, and used for compounds normalization.

### 4.7. Statistical Analysis

The raw MS data were normalized to total ion current (TIC) and statistically analyzed using Perseus ver. 1.5.8.5 [57], and MetaboAnalyst [58]. Data were logarithmized, and sample quality was assessed by calculating the presence of lipids in the samples (cut off was set to 80%). For subsequent analysis, only lipids present across 80% of all samples in each group were retained. In the GC-MS analysis of selected lipid-related compounds, only compounds present in all samples were analyzed (no missing values). Chi-square test was used for categorical variables. Data distribution was assessed using Shapiro–Wilk and Leven’s tests to evaluate the equality of variances. The data were statistically analyzed using a U-Mann–Whitney test or a Student’s unpaired t-test when appropriate. More than two groups were compared using one-way ANOVA or Kruskal–Wallis for nonparametric data with FDR correction, followed by post-hoc multiple comparison testing. Statistical significance was accepted at *p* < 0.05. Particular lipids were considered to be differentially accumulated if apart from the *p*-value < 0.05, the difference between at least 2 groups was at the fold change of ±1.5.

To analyze the average abundance of each lipid class, only *p*-values were taken into account. The correlations between variables were defined by Pearson’s coefficients (for parametric data) or using the Spearman’s rank correlation test (for nonparametric data). A *p*-value of <0.05 was considered statistically significant. Multivariate analyses were carried out by untargeted principal component analysis (PCA) and hierarchical clustering. For hierarchical clustering and heat map visualization, the data were normalized to a Z-score. Partial least squares discriminant analysis (PLS-DA) was additionally performed using the SIMCA software ver. 13.0.0.0 (Umetrics AB, Umeå, Sweden) to check for the potential differences between groups. The data were not split additionally into the training and testing set. Data were log-transformed and Pareto-scaled before the analysis.

## Figures and Tables

**Table 1:** Demographic data and clinical characteristics of the study. Mean value ± SD. *p* < 0.05 was considered to be statistically significant. eGFR—estimated glomerular filtration rate, BMI—body mass index, hsCRP—high-sensitivity C-reactive protein. Chi-square test was used for categorical variables, while for other variables, Mann–Whitney U-test was utilized.

	HV	CKD1-2	CKD5	CVD	* p * -Value
Age [years]	51 ± 16	63 ± 5	61 ± 14	58 ± 10	0.04
Males	75%	75%	75%	75%	0.64
eGFR [ml/min/1.73 m^2^]	102 ± 11	70 ± 6	6 ± 4	96 ± 11	<0.01
BMI [kg/m^2^]	24 ± 2	29 ± 4	25 ± 3	28 ± 4	0.08
Arterial hypertension	0%	100%	100%	100%	<0.01
Glucose [mM]	4 ± 0.5	5.6 ± 0.5	5.8 ± 0.4	5.41 ± 0.6	<0.01
Anticoagulant treatment	0%	79%	54%	87.5%	<0.01
Statin treatment	0%	87.5%	67%	75%	<0.01
Blood pressure treatment	0%	100%	100%	100%	<0.01
Total cholesterol [mg/dL]	190 ± 24	199 ± 46	170 ± 29	187 ± 49	<0.01
HDL cholesterol [mg/dL]	65 ± 16	62 ± 19	44 ± 10	53 ± 13	<0.01
LDL cholesterol [mg/dL]	90 ± 28	131 ± 39	84 ± 27	101 ± 41	<0.01
Triacylglycerols [mg/dL]	101 ± 56	129 ± 66	127 ± 60	101 ± 42	<0.01
hsCRP [mg/L]	1.53 ± 0.4	2.32 ± 2.1	16.67 ± 11	2.17 ± 4.6	<0.01

## Data Availability

The data presented in this study are available on request from the corresponding author. The data are not publicly available due to the specificity and complexity of data sets obtained with shotgun approach.

## Supplementary Materials

The following are available online at https://www.mdpi.com/article/10.3390/metabo11050275/s1, Table S1: Pearson’s correlation coefficients calculated for clinical data, Table S2: A list of identified lipid classes in plasma of HVs, CKD1-2, CKD5 and CVD patients. Table provides information about the name of lipid class, average abundances and SD for all analyzed experimental groups, ANOVA/K–W *p*-value and *q*-value and fold changes for all group comparisons, Table S3: Pearson’s correlation coefficients calculated for identified lipid classes presented as a heat map in Figure 1 and clinical data. The calculations were derived from MetaboAnalyst software. Significant correlation (*p*-value < 0.05) is marked in bold, Table S4: Complete list of differentially accumulated lipid species in plasma of HVs, CKD1-2, CKD5 and CVD patients. Table provides information about the name of lipid class, average abundances and SD for all analyzed experimental groups, ANOVA/K–W *p*-value and *q*-value and fold changes for all group comparisons, Table S5: A detailed list of the enrichment analysis performed in LION. Table provides information about lipid functions, cellular components or lipid classification with term ID, number of annotated lipids, *p*-value and FDR-corrected Kolmogorov–Smirnov *p*-value (*q*-value), Table S6: A list of low-molecular-weight compounds quantified using extracted-ion chromatogram (EIC) and GC-MS analysis. Table provides information about the name of compound, retention time (RT), and *m*/*z* value characteristic for the given compound derived from NIST database (Quant Mass), average abundances and SD for all analyzed experimental groups, ANOVA/K–W *p*-value and *q*-value and fold changes for all group comparisons, Table S7: Spearman’s correlation coefficients calculated for low-molecular-weight compounds quantified using extracted-ion chromatogram (EIC) and GC-MS analysis and clinical data presented in Figure 8, Figure S1: Partial least squares discriminant analysis (PLS-DA) performed on the total number of lipids identified in CKD5 (green), CVD (dark blue), CKD1-2 (red), and HVs (blue) experimental groups.
